# A12 ASPIRIN USE IN PREGNANT PATIENTS WITH INFLAMMATORY BOWEL DISEASE: A SINGLE CENTRE RETROSPECTIVE STUDY

**DOI:** 10.1093/jcag/gwae059.012

**Published:** 2025-02-10

**Authors:** R Jogendran, H Hothi, H Nabavian, S Eisen, V Srikanth, K O’Connor, P Tandon, V Huang

**Affiliations:** Sinai Health, Toronto, ON, Canada; Sinai Health, Toronto, ON, Canada; Sinai Health, Toronto, ON, Canada; Sinai Health, Toronto, ON, Canada; Sinai Health, Toronto, ON, Canada; Sinai Health, Toronto, ON, Canada; University Health Network, Toronto, ON, Canada; Sinai Health, Toronto, ON, Canada

## Abstract

**Background:**

Low-dose aspirin (LDA) prophylaxis is recommended for pregnant patients who are at high risk of developing preeclampsia. Inflammatory bowel disease (IBD) is a risk factor for preeclampsia, however, our understanding of LDA use on disease activity in pregnant patients with IBD remains limited.

**Aims:**

To evaluate the effect of LDA prophylaxis on clinical outcomes and disease activity in pregnant patients with IBD.

**Methods:**

Pregnant patients with IBD followed at Mount Sinai Hospital from January 2017 to December 2022 were identified. We analyzed baseline characteristics and clinical outcomes in patients with and without LDA use. Clinical outcomes included the start date and dose of LDA, obstetric outcomes, and disease activity during pregnancy or 6 months postpartum defined as partial Mayo score ≥ 2 for ulcerative colitis (UC) and Harvey-Bradshaw Index (HBI) ≥ 5 for Crohn’s disease (CD). IBD-related hospitalization, elevated fecal calprotectin ≥ 250 μg/g, or active endoscopic disease were also utilized as markers of disease activity. Bivariate assessments were performed using Chi-Squared tests for categorical predictors. Univariate logistic regression was performed and reported as odds ratios (OR) with 95% confidence intervals (CI).

**Results:**

287 pregnant patients with IBD were included with a median age at conception of 33 years. Forty-three patients (15.0%) were taking LDA during pregnancy, while 244 (85.0%) were not. LDA use was increased in those of older maternal age (34.5 vs. 33 median years, p = 0.047), IBDU (p = 0.016), pre-existing chronic hypertension (7.0% vs. 1.2%, p = 0.016), and prior history of hypertensive disorders during pregnancy (13.9% vs 1.6%, p < 0.001). There was no significant difference with respect to biologic use, disease activity at conception, and parity. Preeclampsia rates were similar between groups (9.3% vs. 6.5%, p = 0.599), although individuals on LDA had higher rates of gestational hypertension (7.0% vs. 0.8%, p = 0.005). There were no differences in IBD flare, elevated fecal calprotectin, and IBD-related hospitalization during pregnancy or postpartum based on LDA use (*P* > 0.05). In univariable logistic regression, active disease at conception (OR 5.66, 95% CI 1.96-16.3, p = 0.001) was associated with an increased risk of flare while the use of biologics was correlated with a decreased risk of flare (OR 0.384, 95% CI 0.238-0.619, p < 0.001).

**Conclusions:**

Low dose aspirin use for prophylaxis of preeclampsia in pregnant patients with IBD did not increase the risk of disease activity. Further studies are required in this patient population to assess the effects of LDA on maternal and neonatal outcomes.

**Funding Agencies:**

None

